# IL-36 receptor antagonist deficiency resulted in delayed wound healing due to excessive recruitment of immune cells

**DOI:** 10.1038/s41598-020-71256-8

**Published:** 2020-09-08

**Authors:** Kenta Saito, Yohei Iwata, Hidehiko Fukushima, Soichiro Watanabe, Yoshihito Tanaka, Yurie Hasegawa, Masashi Akiyama, Kazumitsu Sugiura

**Affiliations:** 1 Department of Dermatology, Fujita Health University School of Medicine, 1-98 Dengakugakubo, Kutsukake-cho, Toyoake, Aichi 470-1192 Japan; 2 Department of Dermatology, Nagoya University Graduate School of Medicine, 65 Tsurumai-cho, Showa-ku, Nagoya, Aichi 466-8550 Japan

**Keywords:** Cytokines, Interleukins, Transforming growth factor beta, Acute inflammation

## Abstract

Loss-of-function homozygous or compound heterozygous mutations in *IL36RN*, which encodes interleukin-36 receptor antagonist (IL-36Ra), have been implicated in the pathogenesis of various skin disorders. Previous findings showed that IL-36γ promoted wound healing in mice; however, the pathogenic role of IL-36Ra in wound healing remains unclear. We elucidated the role of IL-36Ra, a regulator of IL-36 in tissue repair by investigating the recruitment of inflammatory cells and cytokine production in the absence of IL-36Ra. Full-thickness excisional wounds were made on the back of *Il36rn*^−/−^ mice and healing was assessed by monitoring macroscopic wound sizes, numbers of infiltrated cells, and gene expression of inflammatory cytokines. Macroscopic wound healing, re-epithelialization, and granulation tissue formation were delayed by 3 days post-injury in *Il36rn*^−/−^ mice. This delay was associated with increased infiltrations of neutrophils and macrophages, and increased expression of cytokines, such as IL-36γ, C-X-C motif chemokine ligand 1 (CXCL1), and transforming growth factor (TGF)-β. Importantly, administration of TAK-242, a toll-like receptor 4 (TLR4) inhibitor, caused normalization of wound healing in *Il36rn*^−/−^ mice, abrogating the initial delay in tissue repair. These results showed that targeting TLR4- mediated infiltrations of immune cells and cytokine production could be beneficial in regulating wound healing in IL-36Ra-deficient skin disorders.

## Introduction

Interleukin-36 (IL-36) is a member of the IL-1 superfamily, with subfamily members identified as IL-36α, IL-36β, IL-36γ, interleukin-36 receptor antagonist (IL-36Ra) and IL-38^1–3^. Unlike IL-36α, IL-36β, and IL-36γ that act as receptor agonists for pro-inflammatory functions^4^, IL-36Ra is an anti-inflammatory mediator^5^. The *IL36RN* gene encodes IL-36Ra, a protein responsible for the tight regulation of IL-36 signaling. IL-36 related cytokines are increasingly associated with various inflammatory diseases such as inflammatory bowel disease, rheumatoid and psoriatic arthritis, and inflammatory skin disorders. Among the IL-36 associated skin diseases, psoriasis is the most well-known disease.

Responsible for the pathogenesis of psoriasis-related pustular rash is caused by homozygous or compound heterozygous *IL36RN* gene mutations^6–8^. Loss-of-function mutations in *IL36RN* is defined as a recessive hereditary autoinflammatory disease. It was a type of autoinflammatory keratinization disease and called “deficiency of IL-36Ra” (DITRA)^9,10^. In the previous report, *Il36rn*^−/−^ mice was generated and DITRA mouse model was established^11^. Aberrant interleukin-36 receptor (IL-36R) signaling causes transient skin inflammation including hyperkeratosis, acanthosis, and neutrophil-dominant mixed-cell infiltration^11–13^.

According to the Human Genetic Variation Database, two *IL36RN* founder mutations (c.28C > T (p.Arg10X) and c.115 + 6T > C (p.ArgfsX1)) are seen in about 2% of Japanese population^14^. The pathogenic roles of IL-36 in skin inflammatory diseases including atopic dermatitis, blistering disease, and allergic dermatitis have studied^15–17^. We found that deficiency of IL-36Ra increased the contact hypersensitivity response by eliciting excessive infiltration of neutrophils into the skin, a result of the activation of IL-36R-mediated sustained inflammatory signaling^13^. Thus, loss-of function mutations of *IL36RN* may potentially be an underlying cause of various skin inflammatory diseases including generalized pustular psoriasis. Although IL-36γ was shown to promote wound healing in mice^18^, little is known about the pathogenic role of IL-36Ra in the wound healing process. Therefore, we hypothesized that IL-36Ra deficiency may play an important role in wound healing process by regulating proper inflammatory cell recruitment.

Wound healing is one of the most complex biological processes^19^. The infiltration of inflammatory cells is very important in the inflammatory phase of the process. Wound healing is prolonged with excessive or insufficient inflammatory cell infiltration^20–22^. Excessive neutrophil infiltration and increased chemokines and growth factor mRNA expression that resulted in impaired wound healing have been observed in IL-1 receptor antagonist (IL-1ra) deficient mice^20^. Therefore, we hypothesized that IL-36 cytokine may play an important role in wound healing process by regulating proper inflammatory cell recruitment. To verify this hypothesis, we studied the wound healing process in *Il36rn*^−/−^ mice.

## Results

### Deficiency of IL-36Ra caused delayed wound healing and excessive granulation tissue formation

**Figure 1:**
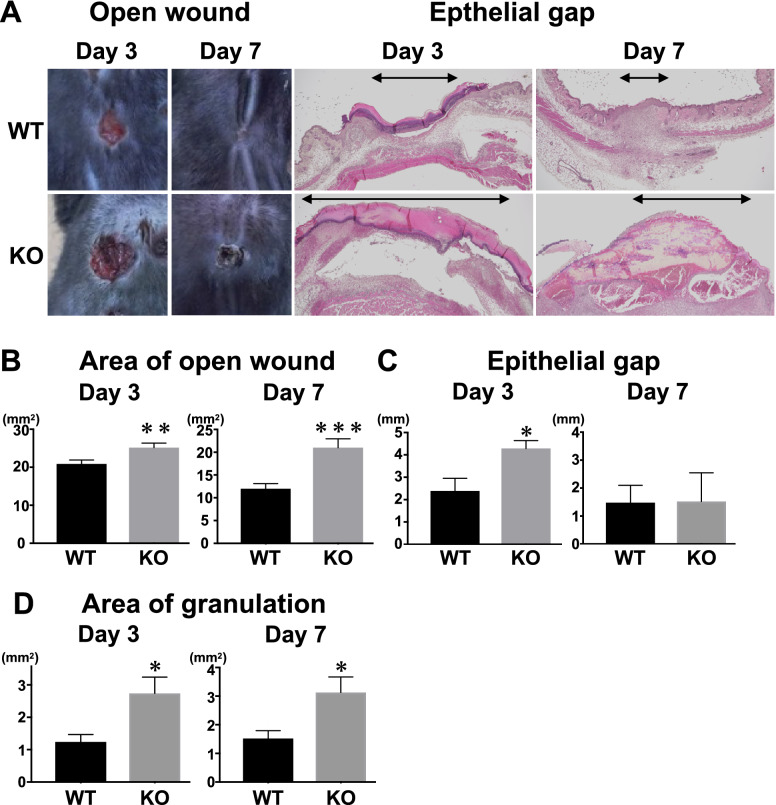
Wound closure and granulation tissue formation in wild type mice (WT), *Il36rn*^−/−^ mice (KO) at 3 and 7 days after wounding. (**A**) Representative photographs of open wounds and histology of wound tissues (epithelial gap and granulation tissue) in WT and KO (magnification original × 40 and × 400). (**B**) The area of open wound was measured at 3 and 7 days after wounding by tracing of the wound openings onto a transparency. The epithelial gap (**C**), which represents the distance between the leading edge of migrating keratinocytes, and the area of granulation tissue (**D**) were both microscopically measured in the tissue sections. We identified areas of newly formed capillaries with a collection of fibroblasts and inflammatory cells as granulation tissues. Each histogram shows the mean ± SEM values obtained from 15 mice (51 wounds) in the WT group, and 15 mice (47 wounds) in KO group (*p < 0.05 versus WT mice).

To assess the effects of deficiency of IL-36Ra on wound healing and tissue repair, areas of open wounds were measured at 3 and 7 days after wounding *Il36rn*^−/−^ and wildtype control mice. At both 3 and 7 days after injury, the open wound area was larger in *Il36rn*^−/−^ mice than in wild type (Fig. 1A,B). We assessed re-epithelialization by measuring the epithelial gap, the distance between the migrating edges of keratinocytes, under the microscope. The epithelial gap was wider in *Il36rn*^−/−^ mice compared with that in wild type mice at 3 days after wounding (Fig. 1A,C). Granulation tissue formation was highly promoted in *Il36rn*^−/−^ mice compared with that in wild type at both 3 and 7 days after wounding (Fig. 1D). Thus, IL-36Ra deficiency led to excessive granulation tissue formation and delayed wound healing.

### Infiltration of inflammatory cells was intensive in wounded *Il36rn*^−/−^ mice

**Figure 2:**
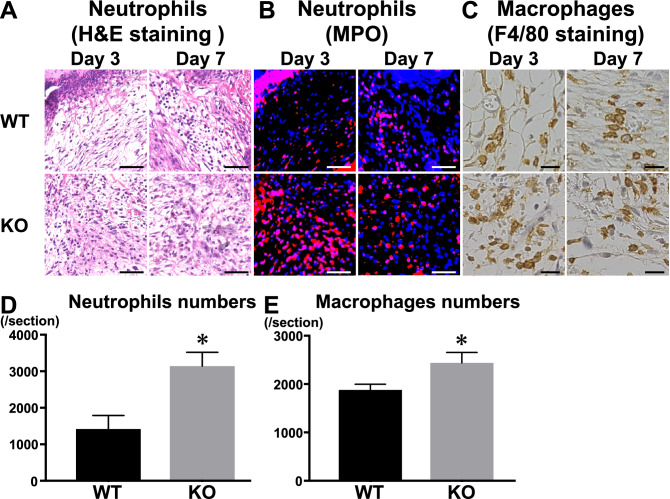
Inflammatory cell recruitment in wounded skin of wild type mice (WT), and *Il36rn*^−/−^ mice (KO) at 3 and 7 days after injury. (**A**–**C**) Representative histological skin sections from WT and KO at day 3 and day 7 after wounding. (**A**) Representative histological of haematoxylin and eosin staining in WT and KO (original magnification, × 100; scale bar 50 μm). (**B**) Immunofluorescence staining of MPO (red) in skin lesions. Nuclei were counterstained with DAPI (blue) (original magnification, × 100; scale bar 50 μm). (**C**) Representative histological of F4/80 staining in WT and KO (× 200; scale bar 20 μm). (**D**) Numbers of neutrophils per section were determined by counting H&E-stained sections under the microscope. (**E**) Numbers of F4/80 staining macrophages per section were also counted under the microscope. Each histogram shows the mean ± SEM values obtained from 10 wounds in each group (*p < 0.05 versus WT mice).

Next, we evaluated infiltration of inflammatory cells into the wound of *Il36rn*^−/−^ mice. The distribution of neutrophils in the lesions of mice was detected by histological haematoxylin and eosin staining and immunofluorescence. Numbers of neutrophils that migrated out of the blood vessels were larger in *Il36rn*^−/−^ mice compared with those in wild type mice at 3 days (*p* < 0.05), but not 7 days (*p* = 0.3) after wounding (Fig. 2A,B,D).

Macrophage numbers were larger in *Il36rn*^−/−^ mice compared with those in wild type mice at 3 days (*p* < 0.05) but not 7 days (*p* = 0.48) after injury (Fig. 2C,E). There was no significant difference in numbers of CD3-positive T cells migrated out of the blood vessels in *Il36rn*^−/−^ mice and wild type mice (data not shown). Collectively, IL-36Ra deficiency resulted in increased infiltration of neutrophils and macrophages into the wound tissues at the early phase of skin injury.

### Cytokine mRNA expression modulated in *Il36rn*^−/−^ mice

**Figure 3:**
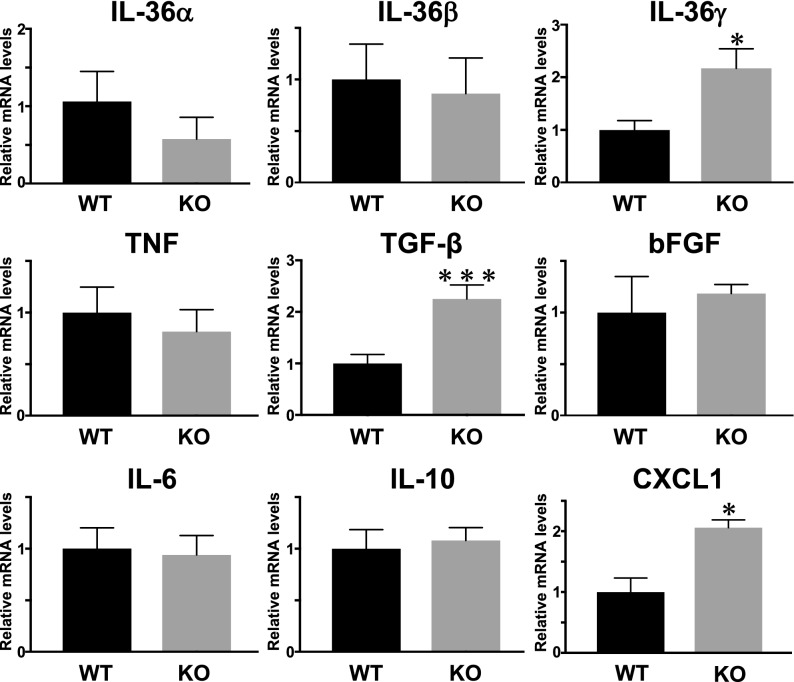
Increase in cytokine expression by IL-36Ra deficiency in the wounded skin. Expression levels of IL-6, IL-10, TNF-α, TGF-β, bFGF, CXCL1, IL-36α, IL-36β, and IL-36γ mRNA wild type mice (WT) and *Il36rn*^−/−^ mice (KO) (n = 7–10; *p < 0.05; ***p < 0.001 versus wild-type mice). GAPDH mRNA was used as an internal control.

Expression levels of IL-36α, IL-36β, IL-36γ, IL-6, IL-10, IL-4, TNF-α, CXCL1, and TGF-β mRNA in the wounded skin tissue were examined by real-time RT-PCR. At 3 days after wounding, *Il36rn*^−/−^ mice showed increased mRNA expression levels of IL-36γ (*p* < 0.05), TGF-β (*p* < 0.001), and CXCL1 (*p* < 0.05) compared with wild type mice (Fig. 3), whereas at 7 days after wounding, there was no significant difference in the mRNA expression between wild type and *Il36rn*^−/−^ mice (data not shown). Thus, IL-36Ra deficiency was considered to increase the expression of IL-36γ, TGF-β, and CXCL1 in skin tissue at 3 days after wounding.

### Hyaluronic acid (HA) accumulated in extracellular matrix of wounded skin

IL-36Ra deficiency resulted in delayed wound healing, enhanced infiltration of neutrophils and macrophages, and an altered mRNA expression of cytokines after 3 days of wounding. These results suggest that IL-36Ra deficiency play a crucial role in early stage of the wound-healing process. Therefore, we hypothesized that IL-36Ra modulate wound-healing through the activation of innate immunity including toll-like receptor (TLR) signal pathways. A recent study demonstrated an essential role of TLR4 in early wound repair^23^. In non-infectious inflammation, certain molecules, called endogenous danger signals, activate TLRs^24–26^. For example, HA binds TLR2 and TLR4 to initiates inflammatory responses and promotes recovery from acute injury^27^. HA is one of the major structural components of the extracellular matrix and undergoes rapid degradation at sites of tissue injury and inflammation^28^.

**Figure 4:**
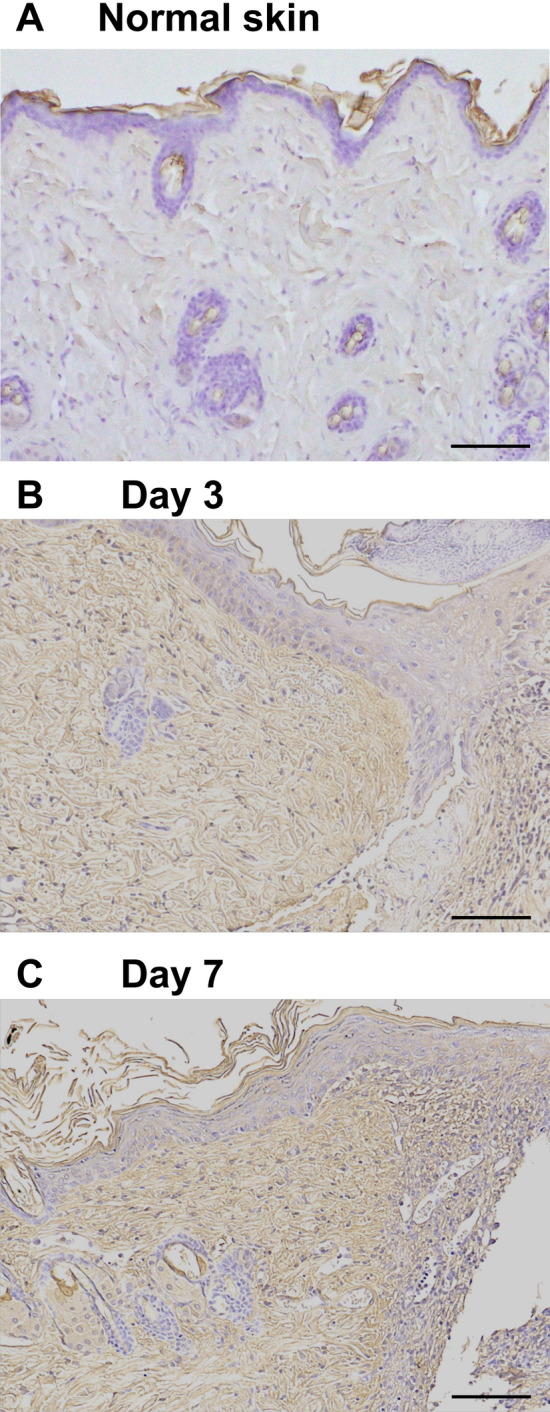
HA accumulation in wild type mice (WT) during wound healing. HA accumulation in normal skin (**A**) and in wounded skin at 3 days (**B**) and 7 days (**C**) after injury was assessed by immunohistochemistry using biotinylated HA binding protein (original magnification, × 100; scale bar 100 μm). No difference was observed in HA accumulation between WT and KO mice.

To examine the contribution of HA to delayed wound healing in *Il36rn*^−/−^ mice, we assessed HA compositions in normal and wounded skin by immunohistochemistry. Although HA was slightly stained in normal skin (Fig. 4A), we found a significant increase in its levels in wounded skins at 3 and 7 days after injury. In addition, HA staining was mainly observed extracellularly (Fig. 4B,C). There was no difference in HA accumulation between wild type and *Il36rn*^−/−^ mice (data not shown).

### IL-36γ mRNA expression and protein levels in HaCaT cells

**Figure 5:**
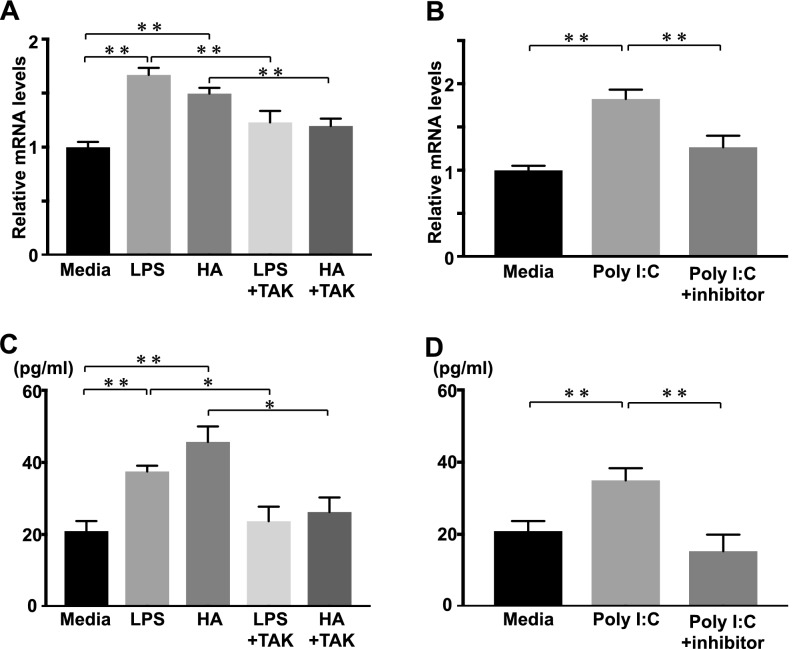
IL-36γ mRNA expression and protein levels in HaCaT cells stimulated with LMW HA and Poly(I:C). Confluent HaCaT cells were stimulated with either media alone (Media), LPS, LWM HA (HA), LPS plus TAK-242 (LPS + TAK) or LMW HA plus TAK-242 (HA + TAK). IL-36γ mRNA expression and protein levels were quantified by real-time RT-PCR and ELISA. Each histogram shows the mean ± SEM values (n = 6; *p < 0.05; **p < 0.01).

IL-36α, IL-36β, and IL36γ can be produced by various cells such as keratinocytes, epithelial cells, monocytes, and lymphocytes^29^. However, keratinocytes are the major source of IL-36γ^30–32^. Therefore, we stimulated HaCaT cells with LPS or low molecular weight HA (LMW HA), and examined IL-36α, β, and γ mRNA expression and protein levels by real-time RT-PCR and ELISA analysis, respectively. Both LPS and LMW HA stimulations significantly increased IL-36γ mRNA expression and protein levels in HaCaT cells, compared with media alone (Fig. 5A,B), while there was no significant change in IL-36α and IL-36β mRNA expression levels (data not shown). The increase in IL-36γ mRNA expression and protein levels in HaCaT cells stimulated with LPS or LMW HA was inhibited by TAK-242, an antagonist of TLR4.

Given that delayed wound healing has been reported in TLR3^−/−^ mice^33^, we also examined IL-36γ mRNA expression and protein levels in HaCaT cells with TLR3 stimulation. TLR3 ligand, Poly(I:C) stimulation increased mRNA expression and protein levels of IL-36γ, and TLR3/dsRNA complex inhibitor suppressed IL-36γ production (Figs. 5C,D). Therefore, HaCaT cells produced IL-36γ by both LMW HA and Poly(I:C) stimulation.

### TGF-β mRNA expression and proteins levels by macrophages

**Figure 6:**
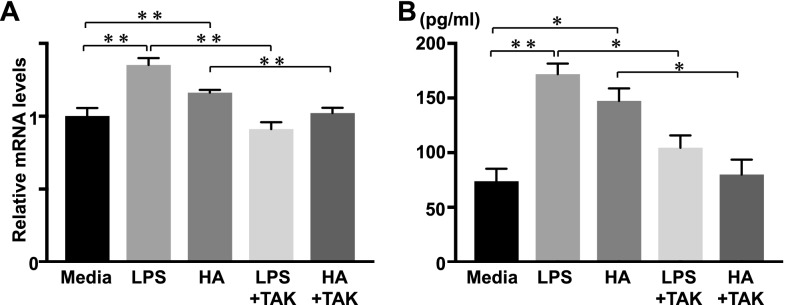
TGF-β mRNA expression and protein levels by macrophage stimulated with LMW HA. Peritoneal macrophages were purified from wild type mice (WT) and stimulated with either media alone (Media), LPS, LMW HA (HA), LPS plus TAK-242 (LPS + TAK) and LMW HA plus TAK-242 (HA + TAK). TGF-β mRNA expression and protein levels were quantified by real-time RT-PCR and ELISA. Each histogram shows the mean ± SEM values (n = 6; **p < 0.01).

We observed an increase in the expression of TGF-β in the wounded skin of *Il36rn*^−/−^ mice at 3 days after injury (Fig. 3). TGF-β is abundant in damaged skin and plays an important role in wound repair^27,34^. Further, HA increases TGF-β expression by stimulating macrophages^21^. We investigated the mechanism of HA stimulation of TGF-β production by HA, which may result in increased infiltration of macrophages in the wound of *Il36rn*^−/−^ mice. Peritoneal macrophages from wild type mice were stimulated with LPS or LMW HA, and TGF-β mRNA expression and protein levels were examined by real-time RT-PCR and ELISA (Fig. 6).

We also examined TGF-β mRNA expression and protein levels in peritoneal macrophages by Poly (I:C) stimulation and found no significant differences (data not shown). Thus, we speculated that low molecular HA increased TGF-β production by peritoneal macrophages via TLR4 signalling.

### Inhibition of HA prohibited wound healing delay in *Il36rn*^−/−^ mice

**Figure 7:**
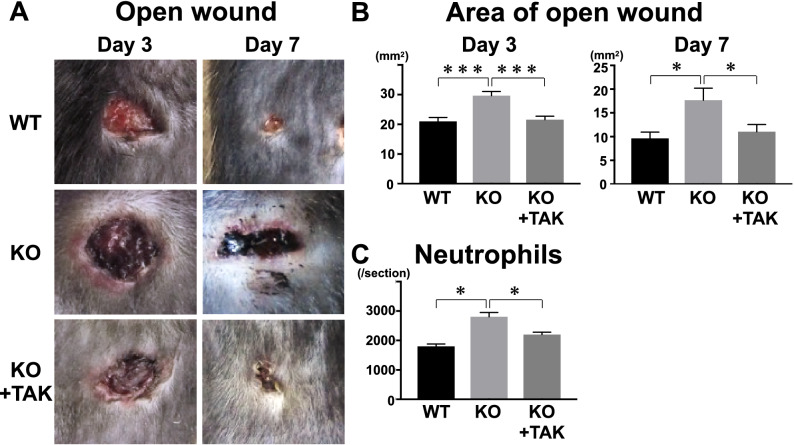
Wound closure in wild type mice (WT), *Il36rn*^−/−^ mice (KO), and KO treated with TAK-242 (KO + TAK) at 3 and 7 days after wounding. (**A**) Representative photographs of open wounds in WT, KO, and TAK. (**B**) Areas of open wound were measured at 3 and 7 days after wounding. (**C**) Numbers of neutrophils at 3 days after wounding per section were determined by counting H&E-stained sections under the microscope. Each histogram shows the mean ± SEM values obtained from 3 mice (12 wounds) in the WT group, and 3 mice (12 wounds) in KO group, and 3 mice (12 wounds) in TAK group (*p < 0.05; ***p < 0.001).

We examined the effect of TAK-242 on delayed wound healing observed in *Il36rn*^−/−^ mice. Intraperitoneal injection of TAK-242 at the early phase of skin injury prohibited the delay in wound healing observed in the control *Il36rn*^−/−^ mice without TAK-242 treatment at 3 and 7 days after injury (Fig. 7).

## Discussion

The current study is the first to demonstrate a critical role of IL-36Ra in the wound healing process. Macroscopic wound healing, re-epithelialization and granulation tissue formation were inhibited due to IL-36Ra deficiency by 3 days after wounding (Fig. 1). Delayed wound healing in *Il36rn*^−/−^ mice was associated with increased infiltration of neutrophils and macrophages (Fig. 2). In addition, *Il36rn*^−/−^ mice also showed increased expression of cytokines, such as IL-36γ, CXCL1, and TGF-β in the skin, especially at 3 days after injury (Fig. 3). In addition, keratinocytes and macrophages were stimulated with HA to release IL-36γ and TGF-β cytokines (Figs. 5, 6). Also, keratinocytes was stimulated with Poly(I:C) to release IL-36γ cytokines (Fig. 5). Taken together, the results of the present study indicate that IL-36Ra deficiency-induced excessive infiltrations of neutrophils and macrophages, and various cytokine production through TLR4 activation, resulted in delayed wound healing.

IL-36 expression induced during tissue damage can modulate immune cells including neutrophils^35–38,52^. In in vitro experiments, IL-36 binds to IL-36R in keratinocytes and macrophages and releases cytokines and chemokines, such as CXCL1, CXCL8, and G-CSF, promoting neutrophil migration^39,40^. Therefore, increased IL-36γ and CXCL1 expression in the injured skin may be reflective of excessive IL-36 and IL-36R signaling on neutrophils, macrophages, and keratinocytes. IL-36 is a member of the IL-1 cytokine family^41^ and IL-1ra deficiency increases inflammatory cell infiltration and delayed wound healing^20^. Since IL-36Ra binds to IL-36R, inhibiting the binding of IL36, and thus exhibiting an anti-inflammatory effect^29^, it is plausible that IL-36Ra deficiency may cause excessive inflammatory cell infiltration, excessive IL-36 and IL-36R signaling, increased cytokine and chemokine release, resulted in delayed wound healing as we observed in our study.

In *Il36rn*^−/−^ mice, delayed wound healing was observed as early as day3; thus, we hypothesized that delayed wound healing was associated with innate immunity, such as TLR4 signaling. This model was in a sterile condition and wound healing occurred without microbial stimuli such as LPS. Therefore, we considered being a candidate that endogenous ligands for TLR4 including HA, HMGB1, and fibrinogen. It has been previously reported that trauma can release small molecular weight HA fragments from the extracellular matrix, thus it may present HA as an endogenous signal of inflammation^24^. Therefore, we focused on HA among the candidates for endogenous ligands in this study. Indeed, we confirmed that HA accumulated in injured skin tissue. HA fragments can signal through TLR4 and/or TLR2, and releasing the proinflammatory cytokines from endothelial cells, macrophages and dendritic cells^19,42,43^. On the other hand, CD44, known as the HA receptor, is expressed on many cells at injured skin^44,45^. Therefore, HA-CD44 signaling may also influence the process of skin wound healing, and to evaluate HA function in *Il36rn*^−/−^ mice, HA-mediated signaling needs to be examined. In the current study, we speculated that the main factor that caused delayed wound healing in *Il36rn*^−/−^ mice would be the different cytokine and chemokine production through TLR4 signaling by IL-36Ra deficiency, and that HA is one of possible candidates for endogenous TLR4 ligand.

We observed increased IL-36γ and TGF-β expression in the wounded skin of *Il36rn*^−/−^ mice. We considered HA, an endogenous ligand for TLR4, as a trigger to induce IL-36γ and TGF-β production in the wounded skin. The concentration of HA has been shown to increase very rapidly in experimental skin wounds and peaks after 3 days of injury^19^, as we confirmed in our study. TLR4 plays an important role in the early stages of wound healing^19,46^. TLR4 is expressed in various cells including keratinocytes and macrophages^47^. TLR4 is important for activation of macrophages via HA stimulation^21,27^. When HA stimulates TLR4 expression in macrophages, it is followed by release of cytokines and chemokines such as TGF-β, CXCL1, CCL20, CCL5, causing neutrophils and macrophages to migrate to the wound site, promoting wound healing^27,48–52^. During these processes, IL-36 is released through the HA-TLR4 pathway^53^. We confirmed that upon stimulation with HA, there was an increase in expression of IL-36γ in HaCaT cells and TGF-β in macrophages. TAK-242, a TLR4 antagonist, effectively decreased HA-induced IL-36γ expression in HaCaT cells. Furthermore, intraperitoneal administration of TAK-242 normalized delayed wound healing in *Il36rn*^−/−^ mice. These previous and current results suggest that TLR4 stimulation by HA increases IL-36γ and TGF-β expression from keratinocytes and macrophages and plays an important role in wound healing.

Delayed wound healing has been reported in TLR3^−/−^ mice^33^. Studies also showed that skin injury increases IL-36γ expression in epidermal keratinocytes, and that TLR3 is required for the induction of IL-36γ^18,54^. Therefore, it is possible that in addition to TLR4, TLR3 signaling may also be involved in delayed wound healing in *Il36rn*^−/−^ mice. In accordance with this possibility, administration of TLR3/dsRNA complex inhibitor showed significant improvement of macroscopic wound closure in *Il36rn*^−/−^ mice (unsubmitted data). In addition, we examined cytokine production by HaCaT cells and macrophages with TLR3 ligand, Poly (I:C). The results showed that in HaCaT cells, Poly(I:C) stimulation increased mRNA expression and protein levels of IL-36γ, and TLR3/dsRNA complex inhibitor suppressed IL-36γ production. By contrast, peritoneal macrophages did not show a significant increase in TGF-β mRNA expression or protein levels by poly(I:C) stimulation (data not shown). Taken together, TLR3 signaling may also be involved in wound healing by a mechanism different from TLR4 signaling. Various additional analysis would be need to elucidate the detailed mechanism by which TLR3 signaling may be involved in Il36rn^−/−^ mice.

We proposed a possible mechanism for the role of IL-36Ra in the wound healing process driven by the HA generated during early days of skin injury. We used a relevant IL-36Ra deficient model to demonstrate that HA stimulated epithelial cells and macrophages via TLR4, causing the release of IL-36 related cytokines and growth factors that promote wound healing by activating other immune cells. IL-36Ra deficiency revealed that IL-36 is a specific activator of IL-36R that may overly induce inflammatory cells in the wounded skin, resulting in delayed wound healing. In addition, since IL-36 is associated with various inflammatory skin diseases such as psoriasis and AD, this result would be a clue to elucidate pathophysiology of various inflammatory skin diseases. The limitations of current study are as follows. First, it remains unknown whether IL-36α or IL-36β is involved in wound healing. Second, the association between increased neutrophil infiltration to the wound and neutrophil extracellular traps (NETs) formation remains unknown in this study. Third, the mechanism of wound healing in a mouse is different from that of humans, primarily due to process of contraction. In murine skin, the extensive subcutaneous striated muscle layer, called the panniculus carnosus, that is absent in the human skin allows the skin to move independently of the deeper muscles, resulting in rapid contraction of skin following wounding^55^. Understanding the contributions of IL-36Ra to the wound repair processes could provide new clues to innovate novel manners for regulation of wound healing.

## Materials and methods

### Animals

*Il36rn*^−/−^ mice were generated as previously reported^11^. All mice were healthy, fertile, and did not display any evidence of infection or disease. All mice were backcrossed between 5 to 10 generations onto the C57BL/6NCr1 (Charles River Laboratories, Inc., Wilmington, Massachusetts, USA) background. For experiments, 8–14 weeks old *Il36rn*^−/−^ mice and age-matched wild-type littermates or C57BL/6NCr1 controls were used. All mice were housed in a specific pathogen-free barrier facility and screened regularly for pathogens.

### Wounding and macroscopic examination

Mice were anaesthetized with medetomidine hydrochloride, midazolam and butorphanol tartrate, and their backs were shaved and wiped with 70% alcohol. Four full-thickness excisional wounds were made per mouse, using a disposable sterile 6-mm biopsy punch (Maruho Co. Ltd, Osaka, Japan), as previously described^34^. At 3 and 7 days after wounding, mice were anaesthetized, and areas of open wounds were measured by tracing the wound openings onto a transparency. No signs suggestive of local infection were detected in the wounded skins. All wounds were macroscopically assessed for wound closure, although wounds that were extremely distorted or whose sizes were difficult to evaluate were excluded. For macroscopic analysis of wound closure, 51 wounds from 15 mice were used in wild type group, and 47 wounds from 15 mice were used in *Il36rn*^−/−^ mice group.

### Histological assessment

Histological assessment of wounds was performed as previously described^34^. Briefly, wounds were harvested and fixed in 3.5% paraformaldehyde and embedded in paraffin. 6-μm paraffin sections were stained with H&E. The epithelial gap, and area of open wound were measured under a light microscope. The number of infiltrated neutrophils and macrophages were counted in the H&E and F4/80 stained sections. All sections were examined independently by two investigators in a blinded fashion.

### Immunohistochemical staining

Immunohistochemical staining of F4/80 and HA was performed as previously described^34^. Briefly, paraffin-embedded sections were stained with rabbit monoclonal antibody (mAb) specific for F4/80 (Cell signal Technology Japan K.K. Tokyo, Japan). HA staining using biotinylated HA binding protein (Hokudo Co. Ltd, Sapporo, Japan) was also performed according to manufacturer’s instructions.

### Immunofluorescence

The neutrophils in the wounded skin tissue were detected by MPO immunofluorescent staining. Immunofluorescent staining was performed as follows: the skin tissues were incubated with primary antibodies against MPO (1:100, R&D Systems Minneapolis, USA) overnight at 4 °C, followed by incubation with fluorescein isothiocyanate-conjugated secondary antibody for 2 h. The nuclei were counterstained with 4′,6-diamidino-2-phenylindole (DAPI) for 5 min.

### Measurement of mRNA expression by real-time RT-PCR

Total RNAs were extracted from wounded skin tissues using Qiagen RNeasy mini kit (QIAGEN, Valencia, CA) and reverse transcribed to cDNA using PrimeScript RT reagent Kit with gDNA Eraser (Perfect Real Time) (TAKARA BIO, Otsu, Japan). Expression levels of IL-6, IL-10, IL-36α, IL-36β, IL-36γ, CXCL1, bFGF, TGF-β, were measured by real-time RT-PCR using the Light Cycler System (F. Hoffmann-La Roche, Ltd, Basel, Switzerland), according to the manufacturer’s instructions. Primers were obtained by pre-validated PrimeTime qPCR Assays (Integrated DNA Technologies, Coralville, IA). GAPDH was used as an internal control. Relative mRNA expression levels were calculated using the 2^−∆∆Ct^ method, as described elsewhere.

### HaCaT cell culture and stimulation

We used immortalized HaCaT cells^56,57^. Cells were cultured in medium containing DMEM, 100 U/mL penicillin–streptomycin, and 10% FBS. For IL-36γ induction experiments, cells were grown to confluency without changing the medium and rendered quiescent by incubation in serum-free DMEM for 1 h. Medium was replaced with fresh DMEM containing LPS (10 μg/ml), LMW HA (500 μg/ml), LPS plus TAK-242 (50 μg/ml), LMW HA plus TAK-242, Poly (I:C) (10 μg/ml), Poly (I:C) plus TLR3/dsRNA complex inhibitor (10 μg/ml) for 4 h. Cells were then harvested for real-time RT-PCR analysis. Furthermore, protein levels of IL-36γ was measured in cells cultured/treated for 72 h by using ELISA kit (Thermo Fisher SCIENTIFIC, Massachusetts, USA) according to manufacturer’s protocol.

### Peritoneal macrophage culture and stimulation

Mouse peritoneal macrophages were harvested by washing the peritoneal cavity with ice cold PBS. The cells were allowed to adhere overnight in DMEM medium supplemented with 10% FBS and 1% penicillin–streptomycin 1% glutamine before use. Macrophages were cultured in serum-free DMEM medium in 24-well flat-bottom plates and stimulated for 6 h with LPS (10 ng/mL), LMW HA (100 μg/ml), LPS plus TAK-242 (10 μg/ml), LMW HA plus TAK-242, Poly(I:C) (10 μg/ml), Poly (I:C) plus TLR3/dsRNA complex inhibitor (10 μg/ml). Cells were then harvested for and real-time RT-PCR analysis. Furthermore, protein expression levels of TGF-β, was measured in cells cultured/treated for 72 h ELISA kit (Thermo Fisher SCIENTIFIC), according to manufacturer’s protocol. Culture cells from unstimulated or stimulated macrophages were harvested. RNA was extracted from peritoneal macrophage with RNeasy Mini Kit (QIAGEN) and analysed for the mRNA level of TGF-β, by real-time RT-PCR.

### TLR4 inhibition with TAK-242

*Il36rn*^−/−^ mice were treated with an intraperitoneal injection of 5 mg/kg TAK-242 or DW daily from Days 0 (the day at wounding) to Day 3 (3 days after wounding). TAK-242 dissolved in DMSO (10 mg/mL) was diluted in DW. Mice were anaesthetized 3 and 7 days after wounding and areas of open wounds were measured by tracing the wound openings onto a transparency, and tissue samples of the wound opening collected for IHC analysis. We analysed 12 wounds from three mice in each group (wild type, *Il36rn*^−/−^, or *Il36rn*^−/−^ mice treated with TAK-242).

### Statistical analysis

Data analysis were performed using GRAPHPAD PRISM software (version 7; GraphPad Software, La Jolla, CA). All data were presented as means ± SEM. Mann–Whitney U test was used to determine the statistical significance of two different samples, and one-way analysis of variance (ANOVA) was used for comparison among multiple experimental groups. Values of *p* < 0.05 were defined as significant.

### Ethics statement

The mice were ethically treated according to the Regulations for the Management of Laboratory Animals at Fujita Health University. All studies and procedures for the ethical use of these animals have been approved by the Animal Care and Use Committee at Fujita Health University (Permit No.: AP16079).

## Abbreviations

IL: Interleukin
IL-36Ra: IL-36 receptor antagonist
CXCL1: C-X-C motif chemokine ligand 1
TGF-β: Transforming growth factor-β
TLR: Toll-like receptor
DITRA: Deficiency of interleukin-36 receptor antagonist
IL-36R: IL-36 receptor
IL-1ra: IL-1 receptor antagonist
TNF-α: Tumour necrosis factor-α
mRNA: Messenger ribonucleic acid
real-time RT-PCR: Real-time reverse transcription-polymerase chain reaction
HA: Hyaluronic acid
LPS: Lipopolysaccharide
CCL: CC chemokine ligand
H&E: Haematoxylin and eosin
PBS: Phosphate buffered saline
mAb: Monoclonal antibody
cDNA: Complementary deoxyribonucleic acid
bFGF: Basic fibroblast growth factor
PCR: Polymerase chain reaction
GAPDH: Glyceraldehyde-3-phosphate
DMEM: Dulbecco's modified eagle's medium
FBS: Foetal bovine serum
PBS: Phosphate buffered saline
DW: Distilled water
SEM: Standard error of the mean
